# Quantitative detection of free 24S-hydroxycholesterol, and 27-hydroxycholesterol from human serum

**DOI:** 10.1186/s12868-014-0137-z

**Published:** 2014-12-24

**Authors:** Veera Venkata Ratnam Bandaru, Norman J Haughey

**Affiliations:** Department of Neurology, Richard T. Johnson Division of Neuroimmunology and Neurological Infections, The Johns Hopkins University School of Medicine, Carnegie 616A, 600 North Wolfe Street, Baltimore, 21287 MD USA; Department of Psychiatry, Division of Geriatric Psychiatry and Neuropsychiatry, The Johns Hopkins University School of Medicine, Baltimore, MD USA

**Keywords:** Alzheimer’s, Multiple sclerosis, HIV associated neurocogntiive disorder, Serum, 24S-hydroxycholesterol, 27-hydroxycholesterol, LC/ESI/MS/MS

## Abstract

**Background:**

Cholesterol metabolism is important for the maintenance of myelin and neuronal membranes in the central nervous system. Blood concentrations of the brain specific cholesterol metabolite 24S-hydroxysterol to the peripheral metabolite 27-hydroxycholesterol may be useful surrogate markers for neurodegenerative diseases including Alzheimer’s disease, Huntington’s disease, HIV-Associated Neurocognitive Disorders, and Multiple Sclerosis. However, current methods to isolate hydroxycholesterols are labor intensive, prone to produce variable extraction efficiencies and do not discriminate between free and esterfied forms of hydroxycholesterols. Since free hydroxycholesterols are the biologically active form of these sterols, separating free from esterfied forms may provide a sensitive measure to identify disease-associated differences in brain sterol metabolism.

**Results:**

We found that average human serum concentrations were 12.3 ± 4.79 ng/ml for free 24(s)-hydroxycholesterol and 17.7 ± 8.5 ng/ml for 27-hydroxycholesterol.

**Conclusion:**

Serum measurements of these biologically active oxysterols may be useful surrogate measures for brain health in a variety of neurodegenerative conditions.

## Background

Hydroxycholesterols are mono-oxygenated derivatives of cholesterol (cholesten-5-3β-ol) that comprise a family of polycyclic compounds that contain a second oxygen atom as a hydroxyl group on the skeleton of cholesterol. *In vivo*, hydroxycholesterols are present as unesterified (biologically active) and esterified forms (largely biologically inert) [1] Hydroxycholesterols are formed through enzymatic conversion of cholesterol or by free radical autoxidation, and exhibit a short half life relative to cholesterol. Hydroxycholesterols are important intermediates in a number of catabolic pathways that regulate a variety of biological effects. For example, hydroxycholesterols are important for cholesterol transport from the periphery to the liver [2], modulate the expression of sterol sensitive genes involved in lipid and sterol biosynthesis [3,4], act as substrates for the formation of bile salts [5], serve as ligands that activate nuclear liver X receptors-α and -β [6], and are involved in the regulation of cholesterol and lipid metabolism and homeostasis [7,8]. In the central nervous system, hydroxycholesterols regulate arachidonic acid release, voltage-gated calcium channels, synaptic plasticity, induce IL-8, promote neurogenesis and induce apoptosis [9-16]. Consistent with these important roles for regulating biological functions, levels of free hydroxycholesterols are extremely low and tightly controlled, with the majority of hydroxycholesterols maintained in esterified forms [17,18].

Many cell types have the ability to oxygenate cholesterol by mechanisms that involve the cytochrome P450 family of oxidases (CYP). Cell type specific expression CYP subtypes results in the tissue-specific production of particular oxysterol species. Several CYP are present in the central nervous system including 24S-hydroxycholesterolhydroxylase (CYP46), a P450 family member that is expressed in neurons, glia and in endothelial cells of the blood–brain barrier [19]. 24S-hydroxycholesterol (Cholest-5-en-3β, 24(*S*)-diol) is the most abundant hydroxycholesterol in brain and is the primary transport form of cholesterol from the central nervous system into the blood, with smaller amounts eliminated through cerebrospinal fluid [20]. It has been suggested by several studies that serum or plasma levels of 24S-hydroxycholesterol may reflect brain developmental and neuropathological changes associated with Alzheimer’s disease (AD), Huntington’s disease and Multiple Sclerosis [21-27]. 24S-hydroxycholesterol is often expressed as a ratio to 27-hydroxycholesterol (25*R*-Cholest-5-en-3β, 26-diol). 27-hydroxycholesterol is formed primarily in the periphery by the P450 enzyme sterol 27-hydroxylase (CYP27) [24]. CYP27 is expressed in arterial endothelium, macrophages and to lesser extents in other tissues such as cortex, spleen, liver, kidney, adrenal gland and heart [28,29]. 27-hydroxycholesterol can function as a ligand for nuclear receptors, liver X receptors (LXR) and farnesoid X-activated receptors (FXRs) [30]. 27-hydroxycholesterol can also regulate hydrocymethylglutaryl-CoA reductase [31,32], and enhances cholesterol efflux from the vascular endothelium [33]. Macrophages have the highest capacity to convert cholesterol to 27-hydroxycholesterol, which is then transported in blood to the liver where it is converted to bile acids [34].

Hydroxycholesterol detection and quantification has been accomplished in a variety of tissues by isotope-dilution gas-chromatography-mass spectrometry (GC-MS) [18,35], gas- and high-performance liquid chromatography (HPLC)/mass spectrometry [36], HPLC with UV detection of cholesterol oxidation products in tissues [37], as ∆4- 3-ketone derivatives by HPLC [38] and as derivatives of GP hydrazones [39]. Because “free” hydroxycholesterol levels are below the detection limits of many instruments, saponification and/or solid phase extraction techniques have typically been used to extract “total” hydroxycholesterols. The primary advantage of these methods is a high yield of hydroxycholesterols. The disadvantages include lengthy sample preparation times, sample loss, inconsistent yields, and the inability to discriminate between free and esterified hydroxycholesterols. In this study we developed a simple and direct extraction protocol and sensitive LC/ESI/MS/MS method for separation and simultaneous quantitative determination of free 24S-hydroxycholesterol and 27-hydroxycholesterol in serum.

## Results

### Identification and Optimization of ESI/MS/MS for 24S-hydroxycholesterol and 27-hydroxycholesterol

**Figure 1:**
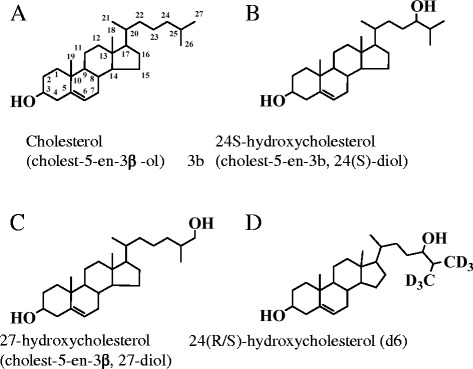
**Structural representation of cholesterol and related products.** Structures of **A)** Cholesterol, **B)** 24S-hydroxycholesterol, **C)** 27-hydroxycholesterol and **D)** 24-(R/S)-hydroxycholesterol (d6).

**Figure 2:**
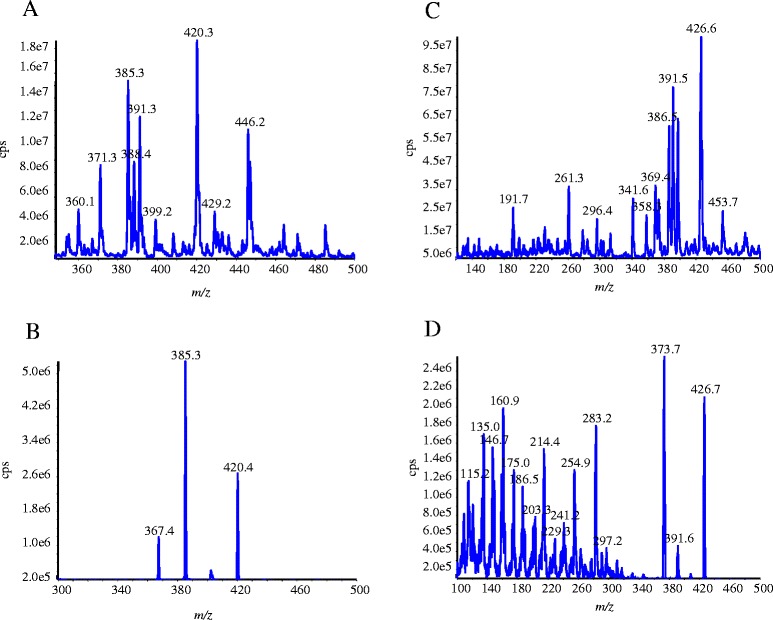
**Identification and fragmentation of 24S or 27-hydroxycholesterol and 24(R/S)-hydroxycholesterol (d6).** Mass spectra for **A)** identification (Q1 scan) and **B)** fragmentation (product ion scan) of a purified 24S or 27-hydroxycholesterol standard (420.3 m/z), **C)** identification (Q1 scan) and **D)** fragmentation (product ion scan) of 24(R/S)-hydroxycholesterol (d6) (426.6 m/z) in positive mode using API3000 mass spectrometer.

The structures of cholesterol, 24S-hydroxycholesterol, 27-hydroxycholesterol and 24(RS)-hydroxycholesterol (d6) are shown in Figure 1. Hydroxycholesterols were identified -as ammonium adducts of 24S-hydroxycholesterol and 27-hydroxycholesterol or 24(R/S)-hydroxycholesterol (d6) (dissolved in pure CH3OH with 5 mM HCOONH4) using ESI/MS operated in the positive ion mode. Molecules of 24S-hydroxycholesterol and 27-hydroxycholesterol were detected as ammonium adducts [M + NH4] with a molecular weight of 420.3 m/z (Figure 2A). The collision induced dissociation of individual hydroxycholesterol species were then characterized by product ion scanning using the highest abundance product ions with adequate signal/noise ratios. For 24S-hydroxycholesterol and 27-hydroxycholesterol the molecular fragment identified was 385.3 m/z (Figure 2B). For 24 (R/S) hydroxycholesterol (d6) the molecular ion identified was 426.6 m/z and the highly abundant product was 373.7 m/z (Figure 2C,D). We optimized the following mass spectrometer parameters that were then used for all hydroxycholesterol species: Ion spray voltage (IS) 2500 eV, temperature (TEM) 250°C, nebulizer gas (NEB) 13 psi, curtain gas (CUR) 8 psi, collision activated dissociation (CAD) 12 psi, dwell time (DW) 150 msec, and entrance potential (EP) 10 eV. The declustering potential (DP), focusing potential (FP), collision energy (CE), collision exit potential (CXP), orientation of the electrospray needle and auxiliary gas flow were individually optimized for each analyte by both direct infusion and flow thorough infusion (FIA) to maximize accuracy and sensitivity (Table 1). We developed our method with two fragmented ions of 24 (R/S) hydroxycholesterol (d6) (426.6/373.7 & 426.7/391.6), and ultimately chose to use 426.6/373.7, as this transition displayed a high signal/noise ratio, a stable low baseline, was abundant, and stable compared to the 426.7/391.6 transition, which showed an unstable baseline (see in Figure 2D).

**Table 1:** **Molecular and fragment ion m/z, and associated parameters for detection and quantification of 24S-hydroxycholesterol, 27-hydroxycholesterol and 24(R/S) hydroxycholesterol (d6) individual species by MRM**

**Anylate**	**Mol. Wt.**	**Molecular ion**	**Fragment ion**	**Time**	DP*	FP*	Dwell*	CE*	CXP*
24S-hydroxycholesterol	402.66	420.3	385.5	10.48	60	400	150	13	5
27-hydroxycholesterol	402.66	420.3	385.5	10.83	60	400	150	13	5
24(R/S) hydroxycholesterol (d6)	408.69	426.4	373.6	10.47	45	250	150	20	10

*DP = Declustereing potential (eV), FP = focusing potential (eV), Dwell = Dwell time (ms), CE = Collision energy (eV), CXP = Collision exit potential (eV).

### Optimization of HPLC conditions for separation of hydroxycholesterols

**Figure 3:**
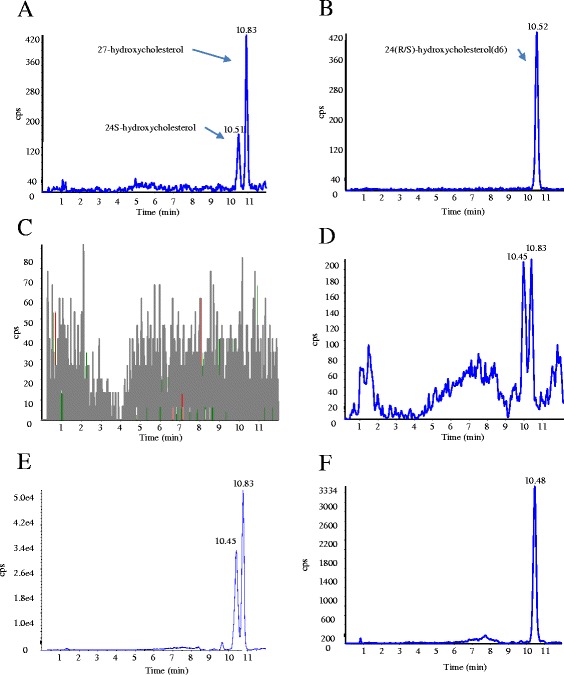
**Chromatograms for 24S-hydroxycholesterol and 27-hydroxycholesterol.** LC/MS/MS chromatograms for **A)** 24S-hydroxycholesterol and 27-hydroxycholesterol and **B)** 24(R/S)-hydroxycholesterol (d6). Base line chromatogram for **C)** methanol. **D)** Chromatogram for 24S-hydroxycholesterol (10.45) and 27-hydroxycholesterol (10.83) extracted from serum. **E)** Chromatogram for 24S-hydroxycholesterol and 27-hydroxycholesterols spiked serum sample. **F)** Chromatogram of 24(R/S)-hydroxycholesterol (d6) added into and extracted from serum sample.

Hydroxycholesterols were separated by HPLC using a C18 column. The HPLC gradient conditions were optimized to obtain good separation between 24S-hydroxycholesterol and 27-hydroxycholesterol with a short running time (~12 min). The best signal to noise separation was observed using pure CH3OH containing 5 mM HCOONH4 as a linear mobile phase. The elution sequence for 24S-hydroxycholesterol, 27-hydroxycholesterol and the internal standard 24(R/S)-hydroxycholesterol (d6) were identified using reference standards (Figure 3). The retention times were 10.45 min for 24S-hydroxycholesterol, 10.83 min for 27-hydroxycholesterol, and 10.48 min for 24(R/S)-hydroxycholesterol (Figure 3A,B).

### Recovery, accuracy and precision

Intra-day and inter-day accuracy and precision were evaluated by spiking known amounts of 24-hydroxycheolsterol, and 27-hydroxycheolsterol (50 ng/ml) in serum (n = 5). The peak area for 24S-hydroxycholesterol and 27-hydroxycholesterol were normalized to the peak area for the internal standard 24(R/S)-hydroxycholesterol (d6). The recovery of 24S-hydroxycholesterol was 59.1% ± 6.99, and recovery of 27-hydroxycholesterol was 55.9% ± 4.77. The inter-day coefficient of variation for 24S-hydroxycholesterol was 8.9% and for 27-hydrosxycholesterol was 3.9%. Intra-day coefficient of variations for 24S-hydroxycholesterol was 11.8%, and for 27-hydrosxycholesterol was 1.9%. Accuracy for 24S-hydroxycholesterol and 27-hydroxycholesterol were between 91 to 118.2% (CVs for 50 and 100 ng doses of 24S-hydroxycholesterol and 27-hydrosxycholesterol are shown in Table 2).

**Table 2:** **Precision and accuracy of hydroxycholesterols**

**Concentration (ng/ml)**	**n**	**Variables**	**hydroxycholesterols**	
			**24S-hydroxycholesterol**	**27-hydroxycholesterol**
			**a. Inter-day analysis**	
50	5	Mean (ng/ml)	50.7	56.9
		SD (ng/ml)	4.5	2.2
		CV (%)	8.9	3.9
		Accuracy (%)	101.4	113.6
100	5	Mean (ng/ml)	91.0	97.5
		SD (μg/ml)	4.7	8.5
		CV (%)	5.1	8.7
		Accuracy (%)	91.0	97.5
			**b. Intra-day analysis**	
50	5	Mean (ng/ml)	49.4	59.0
		SD (ng/ml)	5.8	0.9
		CV (%)	11.8	1.6
		Accuracy (%)	98.8	118.2
100	5	Mean (μg/ml)	98.0	94.9
		SD (ng/ml)	3.3	4.1
		CV (%)	3.4	4.3
		Accuracy (%)	98.0	94.9

n = number of independent replicate, SD = standard deviation and CV = coefficient of variation.

### Linearity, limits of detection and quantification

**Figure 4:**
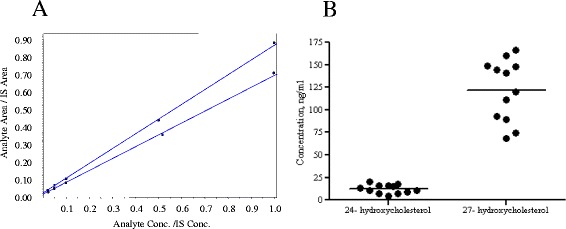
**Quantification of free 24S-hydroxycholesterol and 27-hydroxycholesterol fro** **m normal human serum. A)** Standard curves and **B)** quantities of free 24S-hydroxycholesterol and 27-hydroxycholesterol extracted from serum of healthy volunteers.

Defined amounts of both 24S-hydroxycholesterol and 27-hydroxycholesterol standards (10, 50, 100, 500, 1000 ng/ml) were added to control serum samples prior to extraction. Standard curves were plotted as the ratio of the peak areas for 24S-hydroxycholesterol or 27-hydroxycholesterol to the peak area of the internal standard 24(R/S)-hydroxycholesterol (d6). Least-squares regression analysis for 24S-hydroxycholesterol and 27-hydroxycholesterol standard curves demonstrated linearity in the range of 10 – 1000 ng/ml with a correlation coefficient of r2 for 24S-hydroxycholesterol 0.9979 ± 0.0018 and 0.9940 ± 0.0018 for 27-hydroxycholesterol (Table 3, Figure 4A). The limit of detection was calculated using the signal to noise ratio. The lower detection limits for 24S-hydroxycholesterol and 27-hydroxycholesterol were nearly identical at 248 fmoles on the column. We then calculated the concentration of free 24(S)-hydroxycholeserol and 27-hydroxycholesterol in our human serum samples. The average serum concentrations were 12.3 ± 4.79 ng/ml for 24(s)-hydroxycholesterol and 17.7 ± 8.5 ng/ml for 27-hydroxycholesterol (Figure 4).

**Table 3:** **Linearity calculated by regression analysis from standard curves of hydroxycholesterols**

**Analytes**	^**a**^ ** Regression analysis**				**Correlation coefficient (R)**
	**Slope**		**Intercept**		
	**Mean**	**SD**	**Mean**	**SD**	
24S-hydroxycholesterol	0.3936	0.0988	0.0031	0.0018	0.9979
27-hydroxycholesterol	0.2948	0.0150	0.0127	0.0018	0.9940

^a^ y = m x + c.

## Discussion

The goal of this study was to develop an efficient and rapid extraction protocol for LC/ESI/MS/MS-based detection and quantification of free 24S-hydroxycholesterol and 27-hydroxycholesterol from human serum. For clinical studies, and for the potential use of these hydroxysterols as surrogate markers, it is important that sample analysis be rapid and cost effective. Here we present a simple, inexpensive, and rapid protocol for the extraction of 24S-hydroxycholesterol and 27-hydroxycholesterol from human serum. In addition, the simplicity of the extraction method increases data reproducibility by decreasing variability of product yield. This low cost and rapid sample preparation coupled with the high sensitivity of LC/MS/MS instruments and accurate quantification by MRM make this a potentially powerful approach for the high-throughput quantification of hydroxycholesterol species in clinical and experimental samples.

This extraction method does not use saponification and therefore measures free hydroxycholesterols. This is an important consideration since free hydroxycholesterols are the most biologically active form of these sterols [40,41]. In our healthy human volunteers, serum concentrations ranged from 4 to 21 ng/ml for 24S-hydroxycholesterol and 4 to 29 ng/ml for 27-hydroxycholesterol. These ranges are considerably lower than previously reported levels of total hydroxycholesterol in human serum that range from 60 to 83 ng/ml for 24S-hydroxycholesterol and 120 to 159 ng/ml for 27-hydroxycholesterol [18,24,42,43]. These data are consistent with findings that suggest more than 80% of 24S-hydroxycholesterol and 27-hydroxychoelsterol are maintained in an esterfied state [17].

Since this is the first report of this extraction method, it is not possible to determine if measuring free hydroxycholesterols has a diagnostic or experimental advantage over measuring total hydroxycholesterols. In this study we analyzed a small number of samples to validate the method and did not compare to a disease state. However, we have recently used this method to quantitatively measure levels of free 24S-hydroxycholesterol and 27-hydroxycholesterol in serum of subjects who later developed cognitive impairment, and found that increased levels of free 24S-hydroxycholesterol and the 24S-hydroxycholesterol/total cholesterol ratio were associated with greater risk of impairment on tasks that assess psychomotor speed and executive functioning, while higher levels of free 27-hydroxycholesterol and the 27-hydroxycholesterol/total cholesterol ratio were associated with greater risk of delayed memory impairment. These data were qualitatively different from previous reports that measured total serum levels of these hydroxysterols. For example, total 27-hydroxycholesterol to total cholesterol ratio was associated with a faster decline of immediate memory recall over six years of follow-up [44]. Although, a second reported study did not find an association between total serum 24-hydroxycholesterol or total 27-hydroxycholesterol and cognitive performance [45], this study measured total hydroxysterols. To date, only a single study has directly compared free to total levels of 24S-hydroxycholesterol and 27-hydroxycholesterol in serum. In this study it was reported that 80% of 24S-hydroxycholesterol and 85% of 27-hydroxycholesterol is esterfied in healthy volunteers. Males had higher levels of total 27-hydroxycholesterol compared to females. They found no other demographic or age-related differences in total 24S-hydroxycholesterol or 27-hydroxychoelsterol, and did not determine if there were age- or disease-related differences in free vs. esterfied hydroxycholesterols. Since it is the free forms of these hydroxysterols that have biological activity, and free forms are less than 20% of total hydroxysterols, it is possible that measuring total 24S-hydroxycholesterol and 27-hydroxycholesterol could mask a biological or disease-associated effect. These findings suggest that there may be important differences in free vs. total 24S-hydroxycholesterol and 27-hydroxycholesterols in relation to sex, age and neurodegenerative conditions that merit further study.

The ability to economically and efficiently measure 24S-hydroxycholesterol and 27-hydroxycholesterol in serum may also be useful as surrogate measures for the effectiveness of chemotherapeutics. The approximate cost per sample for this rapid extraction method is $3.00. Saponification with solid phase extraction increases the approximate cost $18.00/sample. These costs are for sample processing and do no include mass spectrometry time that is equal for both methods. Likewise sample run times are 12 min/sample regardless of the extraction method. A number of sterol modifying agents are being tested as potential therapeutics for neurodegenerative disease [46-52], and it is possible that serum measures of 24S-hydroxycholesterol and 27-hydroxycholesterol may have utility as rapid and inexpensive surrogate markers to efficiently determine the effectiveness of therapeutics.

## Conclusion

Serum measurements of these biologically active hydroxycholesterols may be useful surrogate measures for brain health in a variety of neurodegenerative conditions.

## Methods

### Chemicals and equipment

All solvents and chemicals were HPLC grade. Methanol (CH3OH), ethanol (C2H5OH) and diethyl ether (CH3-CH2-O-CH2-CH3) were purchased from Fisher Scientific (USA), ammonium formate (HCOONH4) and formic acid (HCOOH) were purchased from Sigma Aldrich (St. Louis, MO). 24S-hydroxycholesterol and 27-hydroxycholesterol were purchased form Research Plus Inc. (Barnegat, NJ). Internal standard 24(R/S)-hydroxycholesterol (d6) [cholest-5-ene-3β, 24(R/S)-diol (d6)] was purchased from Avanti Polar Lipids (Alabaster, AL). Glass vials were purchased from Agilent Technologies, Inc. (Santa Clara, CA). Isotemp vacuum oven was purchased from Fisher Scientific (Model 285A, Pittsburgh, PA). Borosilicate-coated glass tubes and pipettes were used to reduce adhesion of sterols to plastic and glassware (Fisher Scientific, Pittsburgh, PA).

### Serum samples

Human serum was obtained from 12 healthy volunteers (5 males and 7 females) age 25–36 years at the Johns Hopkins University School of Medicine. Approximately 8 ml (human) blood was collected into BD P100 tubes Sodium heparin (Beckton Dickenson, Franklin Lakes, NJ). Tubes were inverted 8–10 times to mix the protease inhibitors and anticoagulent with the blood sample then placed onto ice. Blood was then centrifuged at 2000 g at 4°C for 15 min. Serum was aliquoted and transferred into cryovials for storage at −80°C until use. All samples underwent a single freeze-thaw cycle before use.

### Extraction of 24S-hydroxycholesterol and 27-hydroxycholesterol

We developed a single step direct extraction method for both 24S-hydroxycholesterol and 27-hydroxycholesterol. For extraction, 0.5 ml of Serum was transferred into a glass tube and 5 μl of 24(R/S)-hydroxycholesterol (d6) (internal standard) from 100 μg/ml stock was added, followed by 3 ml of pure ethanol and the mixture vortexed. Diethyl ether (4 ml) was then were added and the mixture vortexed and centrifuged at 4,000 g for 10 minutes. The supernatant was separated and the residue was re-extracted using the same volumes of solvents as was used in the initial extraction. Supernatants were mixed together, and dried under a stream of nitrogen or in a vacuum oven at 30°C (we did not observe any qualitative or quantitative differences when samples were dried under nitrogen compared with a vacuum oven). Dried extracts were re-suspended into 100 μl of methanol, vortexed, centrifuged and transferred to an autosampler vial insert where it was maintained at 4°C. Samples were injected into the HPLC using an Agilent 1100 series autosampler (Agilent Technologies, Inc., Santa Clara CA, United States).

### Quantification of hydroxycholesterols by LC/ESI/MS/MS using multiple reaction monitoring (MRM)

Sample analysis was performed using triple quadrupole LC/ESI/MS/MS API3000 mass Spectrometer (Applied Biosystems, Thornhill, Ontario, Canada). The HPLC consisted of an Agilent 1100 series with a quaternary pump, degasser, autosampler and thermostatted column. The column was a Luna 5 μM C18 100 Å 100 × 2 mm coupled to a guard column with packing material identical to the column (Phenomenex, Torrance, CA). Chromatography was conducted in gradient elution mode using solvent A (water with 5 mM ammonium formate) and solvent B (pure methanol with 5 mM ammonium formate) at flow rate of 0.3 ml/min. Hydroxycholesterols were separated using the following gradient conditions: 0.0 - 0.3 min, 85% B; 0.3 - 9 min gradient to 100% B; 9–12 min 0% B (Table 4). Injection volume of samples was 10 μl. Quantification was conducted by MRM using Analyst 1.4.2 software (Applied Biosystems).

**Table 4:** **Gradient conditions for LC**

**Step**	**Total time (min)**	**Flow rate (μl/min)**	Mobile phase A* (%)	Mobile phase B* (%)
0	0	300	100	0
1	0.3	300	15	85
2	9	300	0	100
3	12	300	100	0

*A: water with 5 mM ammonium formate, B: pure methanol with 5 mM ammonium formate.

### Standards for quantitative analysis and correction of extraction efficiency

Stock solutions for 24S-hydroxycholesterol, 27-hydroxycholesterol, and 24(R/S)-hydroxycholesterol (d6) (internal standard) were prepared separately in CH3OH to produce a concentration range of 10 ng/ml to 1000 ng/ml. Samples were spiked with 1 μg/ml 24(R/S)-hydroxycholesterol (d6) that was used as an internal control to correct for slight differences in ionization efficiency, chromatographic retention, fragmentation and molecular interactions between hydroxycholesterol species. Blank serum samples were used for background correction. Calibration curves were plotted using the peak area ratios of 24S-hydroxycholesterol or 27-hydroxycholesterol to 24(R/S)-hydroxycholesterol (d6). All stock solutions were stored at −20°C. Calibrations were conducted using least squares linear regression.

#### Standard protocol approvals, registrations, and patient consents

The collection and use of human samples was approved by the IRB the Johns Hopkins University SOM and included written and informed consent for the use of Serum for research purposes.
